# Preschool teachers’ psychological wellbeing, emotion regulation, and emotional responsiveness: a US-Korea comparison

**DOI:** 10.3389/fpsyg.2023.1152557

**Published:** 2023-06-19

**Authors:** Sooyeon Byun, Lieny Jeon

**Affiliations:** ^1^School of Education, Johns Hopkins University, Baltimore, MD, United States; ^2^School of Education and Human Development, University of Virginia, Charlottesville, VA, United States

**Keywords:** early care and education (ECE), teacher wellbeing, emotion regulation, cross-country comparison, teacher responsiveness

## Abstract

**Introduction:**

Psychological wellbeing is an essential indicator of early care and education (ECE) teachers’ positive practices across countries. Moreover, previous studies suggest that teachers’ wellbeing and practice may be indirectly associated via emotion regulation. However, teachers in various contexts demonstrate different patterns of psychological wellbeing, emotion regulation, and emotional responsiveness, and the ways these factors associate with each other also vary.

**Methods:**

The current study investigates whether the indirect associations between ECE teachers’ psychological wellbeing (i.e., emotional exhaustion, job-related competence, and personal stress) and their responsiveness toward children’s emotions via emotion regulation (i.e., reappraisal and suppression) appear differently in two national contexts, the United States (US) and South Korea (SK). Multi-group path analysis was conducted to compare the mediation models between US teachers (*n* = 1,129) and SK teachers (*n* = 322).

**Results:**

We found significant indirect associations among wellbeing, emotion regulation, and responsiveness in both countries. However, significant associations were more prominent among SK teachers, and the patterns of indirect associations had substantial cross-country differences. Furthermore, the roles of reappraisal and suppression emotion regulation found to be different among ECE teachers in SK and US.

**Discussion:**

The cross-country variations in the associations among wellbeing, emotion regulation, and responsiveness suggest that differential policy efforts and intervention strategies are needed for ECE teachers in the US and SK.

## Introduction

Teachers’ wellbeing plays a critical role in creating a classroom environment that promotes positive developmental and learning outcomes among children (Jennings and Greenberg, 2009). The previous literature suggests that multiple features are involved in teachers’ wellbeing in early care and education (ECE) settings, such as burnout, stress, work environments, and health (Cumming and Wong, 2019; Wong et al., 2022). Among these features, this study particularly focuses on the ECE teachers’ psychological wellbeing in their work and personal lives, represented by their emotional exhaustion, job-related competence, and personal stress.

Previous studies conducted in various countries have demonstrated that ECE teachers with high levels of psychological wellbeing tend to create higher-quality classrooms and build more positive relationships with children (e.g., Bae and Moon, 2016; Cassidy et al., 2017; Ansari et al., 2022). Nevertheless, teachers in different national contexts display different levels of psychological wellbeing, and the way that psychological wellbeing associates with teachers’ practice also vary by country (e.g., Aboagye et al., 2018; Hong and Zhang, 2019; OECD, 2020). Theoretically, these cross-country variations attribute to differential social and cultural contexts, such as varied norms, values, behavioral patterns, and policy environment between countries (Jennings and Greenberg, 2009; Gallagher and Roberts, 2022). However, less is known about empirically how and why teachers’ psychological wellbeing and practice are differently associated in ECE settings in different countries.

The purpose of this study is to investigate the cross-country differences in the role of emotion regulation in the association between ECE teachers’ psychological wellbeing and emotional responsiveness in the United States (US) and South Korea (SK). The present study builds upon the authors’ previous study which examined the similarities and differences in the levels of ECE teachers’ psychological wellbeing and responsiveness toward children, and the association between wellbeing and responsiveness in the US and SK (Byun et al., 2022). In this previous study, the authors considered both emotion regulation (internal psychological resources), and emotional exhaustion, job-related competence, and personal stress (indicators of subjective wellbeing), as components of psychological wellbeing. The study found that US teachers, who had more preferrable patterns of emotion regulation (i.e., more reappraisal and less suppression), demonstrated almost no significant associations between emotional exhaustion, job-related competence, and personal stress and responsiveness after accounting for emotion regulation in the model. This is inconsistent with other studies which have found significant associations between educators’ wellbeing and responsiveness (e.g., Jennings, 2015; Jeon et al., 2018). Meanwhile, SK teachers’ emotional exhaustion, job-related competence, and personal stress were significantly associated with responsiveness even after accounting for emotion regulation.

These findings imply that emotion regulation may play a mediating role in the association between teachers’ emotional exhaustion, job-related competence and personal stress and responsiveness. For example, when teachers feel competent in their job and less exhausted or stressed, they may use less positive and more negative emotion regulation strategies, which in turn, would be associated with more desirable responsiveness to children’s emotional expressions. However, teachers may adopt different emotion regulation strategies depending on their contexts, and each emotion regulation strategy may play different roles in different contexts (Matsumoto et al., 2008; Gross, 2014). And such cross-country variations may explain the differences in associations between emotional exhaustion, emotional competence, and personal stress and responsiveness in the US and SK.

Based on this conceptualization, the current study aims to parse the role of emotion regulation and subjective aspects of psychological wellbeing and empirically test the potential indirect associations among these factors. Therefore, in this study, we operationalize psychological wellbeing as the subjective aspects of wellbeing. On the other hand, we examine emotion regulation as a separate feature beyond psychological wellbeing that operates as individuals’ internal psychological resources. Understanding the mechanism of the contextual variations between wellbeing and practice is essential for culturally relevant adaptation of wellbeing interventions and policies in different settings. For example, teachers’ wellbeing and practice may be associated via increased reappraisal in one context and decreased suppression in another context. In this case, teachers in each context may need support on different aspects of emotion regulation to facilitate positive practice. By examining the role of emotion regulation in two different national contexts, the current study expects to better understand the potential mechanisms of cross-country variations in ECE teachers’ wellbeing and its associations with their practice.

In fact, the US and SK have very different cultural backgrounds. Historically, Korea was governed by Confucianism for several hundred years. Due to this background, Korean society is characterized as highly collectivistic, and it values the restraint of gratification and perseverance for the future (often conceptualized as “long-term orientation”) (Hofstede et al., 2010). On the other hand, the US has been influenced by individualistic ideologies, indulgence of gratification, and focus and respect for the past and the present (often conceptualized as “short-term orientation”) (Hofstede et al., 2010). People in individualistic and collectivistic contexts are likely to show significant differences in norms, values, and behaviors (Triandis, 1995). Therefore, simultaneously studying ECE settings in the US and SK will provide insights into potentially different strategies to improve teachers’ wellbeing and practice in different national contexts.

### Relevance of teachers’ emotional responsiveness

In ECE classroom settings, teachers are the first responders to children’s emotional expressions. The literature suggests that teachers respond to children’s emotional expressions either positively or negatively (Jeon et al., 2016; Denham et al., 2017), and this shapes how children’s emotions are socialized (Denham et al., 2012, 2022). With positive responsiveness, teachers help children resolve root problems or sources of negative emotions (*positively-focused reactions*), or acknowledge their negative emotions and encourage them to express the emotions (*expressive encouragement*) (Fabes et al., 1990). Teachers’ positive emotional responsiveness is associated with children’s greater levels of academic and emotional functioning across many national contexts (Bhang and Chung, 2015; Broekhuizen et al., 2017). On the contrary, with negative responsiveness, teachers devalue the seriousness of children’s emotional distress or punish them for negative emotional expressions (*negative reactions*) (Fabes et al., 1990). When teachers use negative responsiveness, their children are likely to experience challenges in social and emotional development (Denham and Bassett, 2019).

Interestingly, the literature shows that the patterns of teachers’ responsiveness to children’s emotions may vary by country. For example, the limited number of US-SK comparison studies have found that US teachers were more accustomed to use positive statements and praise, developmentally appropriate practices, instructional strategies focused on children’s social competence, and relationship-building (McMullen et al., 2005; Clarke-Stewart et al., 2006; Steed et al., 2014; Kim and Han, 2021). Meanwhile, SK teachers tended to use more negative, directive, and teacher-centered instructional strategies with greater focus on skills and drills (Clarke-Stewart et al., 2006; Kim and Stormont, 2012). In Byun et al. (2022), US teachers used positive responsiveness and social guidance (both positive and negative) more frequently than SK teachers, while SK teachers used more negative reactions than US teachers. Cross-cultural differences in teacher practices are common in other cultural contexts as well, such as between the US and Vietnam (Pochtar and Vecchio, 2014), or between the US and China (Mahalingappa et al., 2022).

### Psychological wellbeing and teacher practice

The current study includes emotional exhaustion (helpless, hopeless, and negative feelings about work) (Pines and Aronson, 1988; Maslach et al., 1997), job-related competence (a feeling of mastery and satisfaction about work), and perceived personal stress as indicators of ECE teachers’ psychological wellbeing. Each of these indicators is found to be associated with teachers’ practice in many national contexts. Specifically, when teachers experience increased emotional exhaustion, the quality of their interactions with children tends to be compromised (Kim and Choi, 2018; Ansari et al., 2022). Furthermore, teachers’ increased job-related competence is likely to promote positive teacher practice in the classroom (Cassidy et al., 2017; Kim et al., 2019). Also, ECE teachers with lower levels of stress tend to demonstrate more positive interactions with children (Denham et al., 2017; Seo and Yuh, 2022).

While teachers’ emotional exhaustion, job-related competence, and stress are essential indicators of positive teacher practice across diverse national contexts, the cross-country variation in psychological wellbeing is also a common phenomenon. For instance, a study comparing ECE teachers in Ghana, Pakistan, and China found that teachers in each country encounter differential patterns of psychological challenges (Aboagye et al., 2018). Similarly, in a cross-cultural comparison study between Chinese and Norwegian ECE teachers, Chinese teachers reported more severe challenges related to their work, which in turn, was associated with greater emotional pressure than Norwegian teachers (Hong and Zhang, 2019).

In the previous study by Byun et al. (2022), SK teachers reported lower levels of psychological wellbeing than US teachers, such as significantly lower job competence and greater personal stress. Moreover, psychological wellbeing was more strongly associated with teachers’ responsiveness among SK teachers than US teachers. Notably, US teachers in Byun et al. (2022) demonstrated no significant association between personal stress and responsiveness, unlike other studies that reported significant associations between stress and teacher practice (Whitaker et al., 2015; Denham et al., 2017; Jeon et al., 2018). The key difference was that Byun et al. (2022) accounted for emotion regulation by including it as one of the indicators of teachers’ psychological wellbeing, whereas other studies did not include emotion regulation in the model. This finding indicates that emotion regulation might serve as a distinctive mediator in the association between teachers’ psychological wellbeing and responsiveness. Still, even after accounting for emotion regulation, the association between personal stress and responsiveness was significant among SK teachers. This cross-country difference may be partially explained by differential levels of psychological wellbeing, emotion regulation, and responsiveness among teachers in the two countries. However, it may also be the case that emotion regulation plays distinct roles in teachers in two countries, differing the associations between wellbeing and responsiveness in each context. Inspired by this previous study, the current study is designed to understand why and how psychological wellbeing is differently associated with teachers’ responsiveness across different national contexts.

### Role of emotion regulation

Emotion regulation is defined as “the processes by which individuals influence which emotions they have, when they have them, and how they experience or express these emotions” (Gross, 1998, p. 275). Emotion regulation allows people to modify the process of emotion generation, the trajectory of emotions, and experiences of emotions such as timing, duration, and magnitude (Gross, 2014). McRae and Gross (2020) suggests that there are five ways of administering emotion regulation. People can (a) select situations that will provide desired emotion experiences, (b) modify the situation to make it provide desired emotion experiences, (c) manage deployment of their attention to control emotion experiences, (d) cognitively change the way that they appraise the situation to control emotion experiences, and (e) modulate the way of reacting to emotions so that they can ultimately influence emotion experiences.

Among these five strategies, reappraisal emotion regulation, as an example of cognitive change, and suppression emotion regulation, as an example of response modulation, are the most well-studied emotion regulation strategies (Gross, 2014). Reappraisal emotion regulation changes one’s cognitive evaluation or understanding of the cause of emotions, whereas suppression emotion regulation is direct restraint of one’s reaction to emotions (Gross, 2002). In general, reappraisal emotion regulation is considered to bring more preferrable social, emotional, and cognitive outcomes than suppression emotion regulation, although the consequences of each emotion regulation strategy can be highly context-dependent (McRae and Gross, 2020).

In the education setting, teachers use various emotion regulation strategies when they interact with children. Particularly, in the face of children’s challenging behaviors, teachers most frequently used direct modulation of emotions, such as suppression emotion regulation, along with attention deployment and reappraisal (Chang and Taxer, 2021). Previous studies indicate that, across various countries, there are significant associations between emotion regulation and teachers’ practices in ECE settings as well (Jeon et al., 2016; Karabay, 2019; Jeong and Lew, 2021). In the US, teachers’ use of reappraisal emotion regulation was related to their healthy reactions to children, while using suppression emotion regulation was associated with undesirable ways of responses (Jeon et al., 2016). A Turkish study also identified a significant association between teachers’ reappraisal emotion regulation and high-quality teacher-child relationships (Karabay, 2019). In SK, consistent with studies from other countries, ECE teachers’ emotion regulation was associated with higher self-esteem and positive teacher-child interactions (Jeong and Lew, 2021).

In addition, emotion regulation may mediate the association between teachers’ psychological wellbeing and practice in ECE settings. A study on Italian middle and high school teachers demonstrated that, when teachers’ needs were satisfied, they were likely to use increased reappraisal emotion regulation, which in turn, was associated with autonomy-supportive and motivating teaching styles (Moè and Katz, 2021). Although there is no previous study examining the mediating role of emotion regulation among ECE teachers, we expect to find similar trends in ECE settings, too.

Sheppes (2014) suggests that healthy individuals are likely to account for costs and benefits when they choose different emotion regulation strategies. Theoretically, suppression emotion regulation, which restrains the emotional expression while emotions are being experienced, consumes more cognitive resources than reappraisal emotion regulation, which changes perspectives or mindsets to eliminate undesirable emotions (Gross, 2002). Therefore, it is reasonable to assume that people in a healthier psychological state (i.e., higher level of psychological wellbeing) will use more reappraisal emotion regulation than suppression emotion regulation. On the contrary, when individuals are under psychological challenges (i.e., lower levels of psychological wellbeing), they may have reduced cognitive capacity which can result in less logical decision-making (e.g., Cibrian-Llanderal et al., 2018), such as using emotion regulation strategies consuming more cognitive resources. As a result, teachers with higher levels of psychological wellbeing may use healthier emotion regulation strategies, which in turn, may be associated with more desirable responsiveness toward children’s emotions. Chang and Taxer (2021) study on US middle school teachers provides empirical evidence. In this study, the authors found that teachers tend to use high levels of reappraisal and low levels of suppression when they perceive the low levels of anger, emotional exhaustion, and feelings of challenge, and a high level of enjoyment in teaching (Chang and Taxer, 2021).

However, individuals potentially demonstrate different emotion regulation strategies depending on their social contexts and situational norms (Matsumoto et al., 2008; McRae et al., 2011; Ma et al., 2018). Mesquita et al. (2014) suggest that every aspect of emotion regulation can be influenced by the cultural contexts surrounding individuals. According to the authors, what is considered as *right emotions*, and the way people interpret situations and express emotions, are highly dependent on cultural backgrounds. Thus, individuals are accustomed to regulate their emotions to match what is expected in their cultural context (Mesquita et al., 2014). People in countries putting high emphasis on the traditional social order, illustrated by collectivism and the restraint of gratification (e.g., SK; Hofstede et al., 2010), tend to perceive emotions as interpersonal and relational features (Hochschild, 1995; Gross, 2007). On the contrary, people in countries with the opposite characteristics, such as individualism and indulgence of gratification (e.g., the US) are likely to consider emotions as individual features (De Leersnyder et al., 2013). Given that ECE teachers’ practice is interpersonal and relational by its nature, the discrepancy in the conceptualization of emotion between the US and SK can influence the way teachers regulate emotions in each context, and how it relates to the association between ECE teachers’ psychological wellbeing and their responsiveness toward children’s emotions.

### The present study

Although previous studies have demonstrated significant associations between various wellbeing indicators and teacher practice, to our knowledge, no studies have examined their indirect associations via emotion regulation. In addition, no studies have examined how these indirect associations may vary by national contexts. The current study attempts to fill this knowledge gap by investigating cross-country variations between SK and the US in the associations among psychological wellbeing, emotion regulation, and emotional responsiveness.

Specifically, we aim to answer the following research questions: (1) Is teachers’ psychological wellbeing indirectly associated with more positive and less negative responsiveness toward children via emotional regulation in SK and the US? In other words, is teachers’ psychological wellbeing associated with their emotion regulation, which in turn, associated with their responsiveness toward children?; and (2) Are there any cross-country variations between SK and the US in these indirect associations? We hypothesize that teachers’ psychological wellbeing (i.e., higher levels of job-related competence, and lower levels of emotional exhaustion and stress) would be significantly associated with their use of healthier emotion regulation strategies (i.e., higher levels of reappraisal and lower levels of suppression), which then would be associated with more positive and less negative responsiveness toward children. We also hypothesize that there would be substantial cross-country variations in the strengths and patterns of the indirect associations.

## Methods

### Participants

A total of 1,451 teachers from private and public ECE centers participated in the study, composed of 1,129 US teachers and 322 SK teachers. Demographic and professional characteristics of the participants are presented in Table 1. Most of the participants were female. Sixty-seven percent of US teachers and 72% of SK teachers held a bachelor’s degree or higher. The participants’ mean age was 44.52 years old (*SD* = 12.32) in the US, and 32.89 years old (*SD* = 8.48) in SK. The average years of experience in the ECE field was about twice as high for US teachers (*M* = 15.56 years, *SD* = 9.62) than for SK teachers (*M* = 7.67 years, *SD* = 6.38). Among the US sample, 85% were White, non-Hispanic, 8% were Black, non-Hispanic, 2% were Hispanic, and 5% were other races. We did not collect race/ethnicity data in SK because SK is considered to be an ethnically homogeneous country.

**Table 1:** Descriptive statistics for study variables by country.

Variable name	US		SK		*t*
	N	*M* (*SD*)/%	N	*M* (*SD*)/%	
Responsiveness					
Positively-focused reactions	1,129	5.77 (0.88)	322	5.48 (0.91)	5.09***
Expressive encouragement	1,096	5.31 (1.15)	315	5.01 (1.00)	4.22***
Negative reactions	1,059	1.36 (0.63)	305	2.76 (1.04)	−28.84***
Emotion regulation					
Reappraisal emotion regulation	1,116	5.46 (0.97)	321	5.04 (1.00)	6.64***
Suppression emotion regulation^a^	1,114	3.24 (1.17)	320	3.64 (1.25)	−5.27***
Psychological wellbeing					
Emotional exhaustion	1,119	3.53 (1.68)	322	3.67 (1.23)	−1.41
“I am emotionally exhausted by my work”	1,118	3.44 (1.83)	321	4.17 (1.78)	−6.29***
“Dealing with children’s behaviors drains my energy”	1,117	3.62 (1.75)	317	3.17 (1.20)	4.29***
Job-related competence	1,109	6.29 (1.04)	321	4.86 (1.28)	20.61***
Personal stress	1,129	11.97 (5.66)	319	18.13 (4.68)	−17.82***
Covariates					
Job satisfaction	1,120	6.23 (1.09)	322	5.00 (1.33)	16.99***
Disciplinary self-efficacy	1,120	4.26 (0.62)	321	3.78 (0.68)	11.99***
Years in ECE field	1,114	15.56 (9.62)	310	7.67 (6.38)	13.63***
BA degree or higher	1,109	67%	322	72%	−1.80
Perceived health condition	1,112	4.01 (0.78)	322	2.74 (0.96)	24.42***
Age	1,062	44.52 (12.32)	312	32.89 (8.48)	15.62***
Female	1,110	97%	320	97%	0.60

Adapted from Byun et al. (2022).

^a^Higher scores refer to lower suppression emotion regulation.

***p < 0.001.

Missing data ranged from 0 to 6%. Specifically, in the US sample, there were 0–6.2% of missing data for teacher responsiveness, 0–1.77% for psychological wellbeing, 1.15–1.33% for emotion regulation, and 0.8–5.9% for covariates. For the SK sample, there were 0–5.9% of missing data for teacher responsiveness, 0–0.93% for psychological wellbeing, 0.31–0.62% for emotion regulation, and 0–3.73% for covariates.

### Procedures

The study was approved by the Johns Hopkins University’s Institutional Review Board (IRB). For both US and SK samples, respondents were informed that, by completing the survey, they were considered to provide consent to participate in the survey. The US teacher data were collected in 2014. The research team randomly selected 7,500 center-based ECE facilities in 50 states using the proportional stratified random sampling based on program type (private vs. public) and geographic regions. A survey packet was mailed to the director of each program. In each packet, the researchers included a $1 bill as an incentive, and sealed packets with the teacher survey inside so that teachers could individually mail the survey back to the research team. A reminder post card was sent out 3 weeks after the initial survey distribution. Among 7,500 contacted programs, 455 programs either had an undeliverable addresses or no qualified teachers to participate. The final sample of 1,129 teachers (16% response rate) was not statistically different from the original proportion of program types and geographic regions that were used for stratified random sampling.

The SK teacher data were collected in 2019. Snowball sampling was used to recruit public and private ECE programs in Seoul, Gyeonggi-do, and Gangwon-do regions. Although the research team is based in the US, two members of the research team visited SK for the effective recruitment and data collection. We included the same measures that were used in the US survey for assessing our key variables in the SK teacher survey. However, some of the items on demographic and professional background had to be modified based on SK ECE context, based on the feedback from an ECE expert in SK. When measures had official Korean versions, or had been translated in the previous research, we used those versions; otherwise, measures were translated by an undergraduate research assistant, reviewed by research team members who are fluent in Korean, and an ECE scholar in SK, and finally certified by a third person whose first language is Korean. The research team mailed the survey to each participating ECE program, with sealed envelopes for teachers to return them anonymously. Each program collected completed teacher surveys and sent them back to the research team. Among 375 initially distributed surveys, 322 surveys were returned with valid data for the data analysis (86% response rate).

### Measures

#### Emotional responsiveness

Positively-focused reactions, expressive encouragement, and negative reactions subscales were adapted from the shortened version of the Coping with Children’s Negative Emotions Scale (CCNES; Fabes et al., 1990). These three subscales, originally developed for parents, were validated in a previous study with ECE teachers (Buettner et al., 2016). CCNES presents seven scenarios in which children experience negative emotions or challenging social interactions, and ask teachers to rate their likelihood to respond to each scenario in each of multiple different ways using a 7-point Likert scale (1 = *Very Unlikely*, 7 = *Very Likely*). In this study, Cronbach’s alphas were 0.78 for positively-focused reactions, 0.75 for expressive encouragement, and 0.80 for negative reactions.

#### Psychological wellbeing

##### Emotional exhaustion

Two items were adapted from Buettner et al. (2016) to assess teachers’ emotional exhaustion associated with their job and children’s behaviors using a 7-point Likert scale (1 = *Strongly disagree*, 7 = *Strongly agree*). The 2 items were averaged to produce a composite score for emotional exhaustion.

##### Job-related competence

To measure job-related competence, teachers were asked whether they “feel competent in their job,” using a 7-point Likert scale (1 = *Strongly disagree*, 7 = *Strongly agree*). The raw score was used as a variable representing teachers’ job-related competence.

#### Personal stress

The Perceived Stress Scale (PSS) (Cohen et al., 1983; Park and Seo, 2010) was used to assess teachers’ perceived stress. The scale is composed of 10 items asking about levels of perceived stress during the past month. Teachers were asked to respond on a 5-point Likert scale (1 = *Never*, 5 = *Very Often*) for each item, and the summed score was used as a composite score of teachers’ perceived stress (Cronbach’s alpha = 0.82).

#### Emotion regulation

Reappraisal and suppression emotion regulation were measured by the Emotion Regulation Questionnaire (ERQ) (Gross, 1998). ERQ is a 10-item questionnaire composed of 2 subscales measuring cognitive reappraisal and expressive suppression. Each item used a 7-point scale (1 = *Strongly disagree*, 7 = *Strongly agree*), and the average scores were used as composites scores. Cronbach’s alphas were 0.78 for reappraisal and 0.77 for suppression.

#### Covariates

Seven covariates were included in the model. Teachers’ job satisfaction was measured by 2 items asking about how teachers are satisfied with being an early childhood teacher, and with one’s current position (Buettner et al., 2016). Teachers’ disciplinary self-efficacy was assessed by the Teacher Self-Efficacy Scale (Bandura, 1997) (Cronbach’s alpha = 0.81). Teachers’ years of ECE experience, educational attainment (1 = *a bachelor’s degree or higher*, 0 = *less than a bachelor’s degree*), age in years, and sex (1 = *female*, 0 = *male*) were also examined. Teachers’ overall perceived health condition was assessed by an item from the Respondent-Assessed Health Status Scale (Adams et al., 2012), using a 5-point Likert scale (1 = *excellent*, 5 = *poor*).

### Analytic strategy

Descriptive statistics and pairwise correlations were used to explore the characteristics of study variables in each country. A multi-group path analysis was conducted using Stata 17.0 to simultaneously test the direct and indirect associations between psychological wellbeing and responsiveness via two emotion regulation subscales, and to examine the differences in the models between US and SK samples. Although direct associations among key study variables were reported in Byun et al. (2022), we explored findings on direct associations in the current study as well, to provide a comprehensive picture.

To simulate the distribution of indirect associations, 5,000 bootstrapped samples were used (Kline, 2016). The significance of the indirect associations were estimated using a Sobel test (Sobel, 1982). Multiple model fit indices were used to examine the overall model fit: chi-square test (*χ^2^*) (expected to be nonsignificant, *p* > 0.05), a root mean square error of approximation (RMSEA) (expected to be less than 0.05), the Tucker–Lewis index (TLI) (expected to be greater than 0.95), and a comparative fit index (CFI) (expected to be greater than 0.95) (Browne and Cudeck, 1993; Hu and Bentler, 1999). Among US teachers, having missing values was associated with having higher levels of positively focused reactions, lower levels of stress, more years of ECE experiences, less than a bachelor’s degree, and older ages. In Korea, having missing values was associated with higher levels of expressive encouragement. We assumed missing at random, which means that missingness can be explained by the observed variables within the study, and the Full Information Maximum Likelihood (FIML) estimation was used to handle missing data, preserving relationships among all available data (Arbuckle, 1996).

To compare the statistical differences in the indirect associations between US and SK samples, the structural invariance of the hypothesized associations between US and SK teachers were tested using a multi-group analysis, with country as the grouping variable (Kline, 2016). For the between-group comparison for each parameter, the delta method was used (StataCorp, 2021). If the test statistics are not significant, this indicates that the parameter is invariant between groups, otherwise, the parameters are significantly different between groups (Kline, 2016).

## Results

### Descriptive statistics and bivariate correlations

Descriptive statistics among key study variables are presented in Table 1. Overall, US teachers had higher levels of psychological wellbeing, healthier emotion regulation strategies (more reappraisal and less suppression), and more desirable patterns of emotional responsiveness (more positive responsiveness and less negative responsiveness) than SK teachers. Teachers from both countries demonstrated significant correlations among study variables. The full correlation matrix for each country can be found in Table 2.

**Table 2:** Correlations among study variables by country.

	1.	2.	3.	4.	5.	6.	7.	8.
1. Positively-focused reactions	–	0.55***	−0.02	0.33***	−0.00	0.03	0.13*	−0.20***
2. Expressive encouragement	0.37***	–	−0.06	0.32***	−0.06	−0.04	0.16**	−0.23***
3. Negative reactions	−0.18***	−0.16***	–	−0.17**	0.28***	0.29***	−0.19***	0.31***
4. Reappraisal emotion regulation	0.20***	0.21***	0.00	–	0.14*	−0.08	0.33***	−0.30***
5. Suppression emotion regulation^a^	0.04	−0.07*	0.18***	0.09**	–	0.23***	−0.06	0.21***
6. Emotional exhaustion	−0.04	0.02	0.03	−0.08**	0.09**	–	−0.20***	0.46***
7. Job-related competence	0.09**	0.08**	−0.07*	0.15***	−0.13***	−0.08**	–	−0.32***
8. Personal stress	−0.07*	−0.03	0.08*	−0.16***	0.10**	0.38***	−0.25***	–

Adapted from Byun et al. (2022). Correlations for the US sample are presented below the diagonal and correlations for the SK sample are presented above the diagonal.

^a^Higher scores refer to lower suppression emotion regulation.

*p < 0.05, **p < 0.01, ***p < 0.001.

### Multigroup path analysis

**Figure 1:**
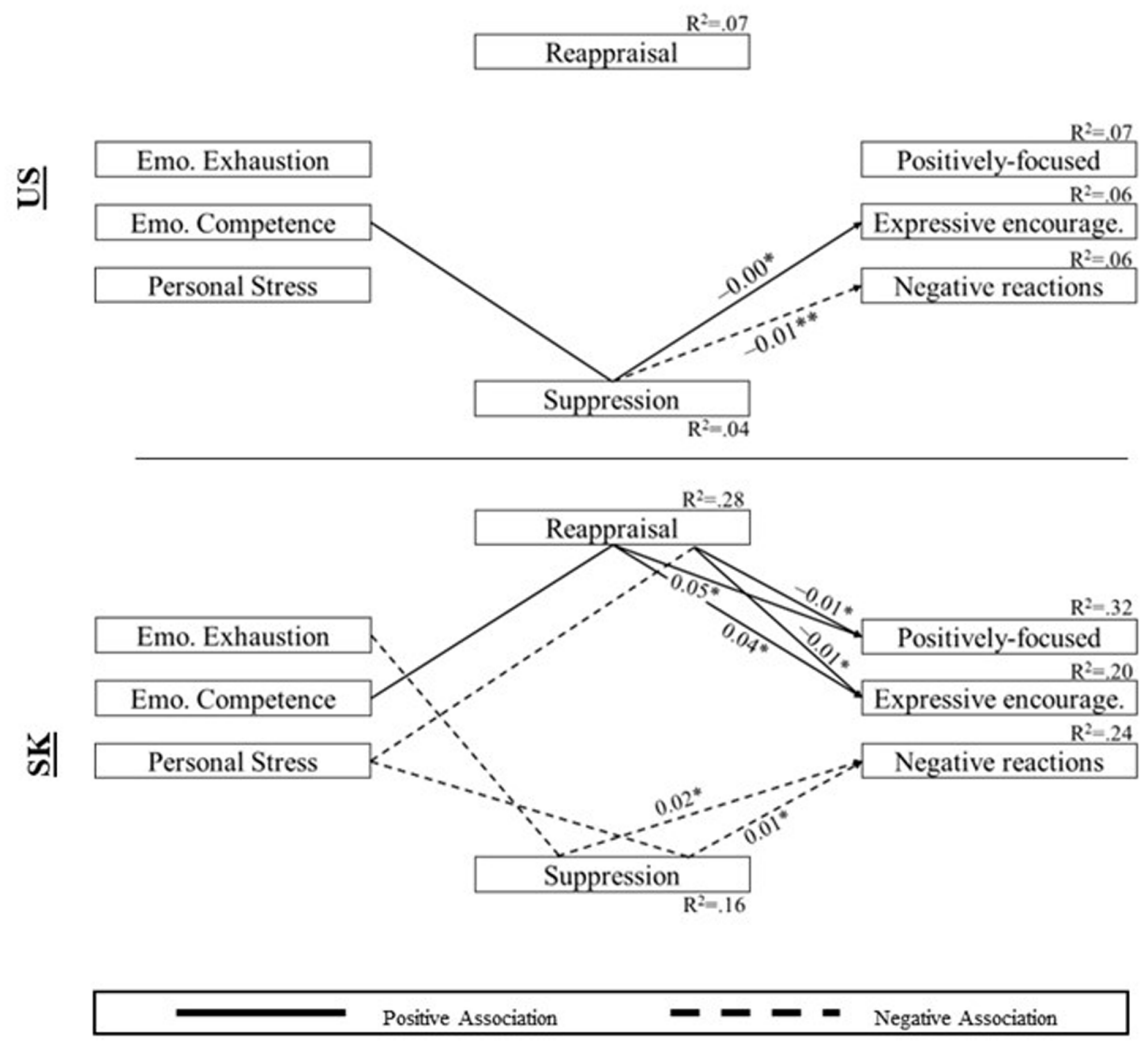
Indirect associations among study variables. Unstandardized coefficients for significant indirect associations are presented. Paths for non-significant associations and covariates, and coefficients for direct associations are omitted. *p < 0.05, **p < 0.01.

The model allowing all parameters to be freely estimated indicated a good fit, *χ*^2^(22) = 19.31, *p* = 0.63, RMSEA = 0.000 [0.000, 0.027], TLI = 1.015, CFI = 1.000. For US teachers, the model explained 7.3% of the variance in positively-focused reactions, 6.4% of the variance in expressive encouragement, 5.6% of the variance in negative reactions, 7.3% of the variance in reappraisal emotion regulation, and 4.4% of the variance in suppression emotion regulation. For SK teachers, the model explained 31.9% of the variance in positively-focused reactions, 19.9% of the variance in expressive encouragement, 24.2% of the variance in negative reactions, 27.5% of the variance in reappraisal emotion regulation, and 15.5% of the variance in suppression emotion regulation. The path analysis coefficients for full models can be found in Table 3. Total, direct, and indirect associations are presented in Table 4 and Figure 1.

**Table 3:** Multigroup path analysis.

	Positively-focused reactions							Expressive encouragement						
	US			SK			Multi-group	US			SK			Multi-group
	B	SE	*β*	B	SE	*β*		B	SE	*β*	B	SE	*β*	
Mediators														
Reappraisal	0.15***	0.03	0.16	0.34***	0.05	0.37	−3.24**	0.27***	0.04	0.23	0.28***	0.06	0.28	ns
Suppression^a^	−0.01	0.02	−0.02	0.05	0.04	0.07	ns	0.09**	0.03	0.09	0.10	0.05	0.12	ns
Psych. wellbeing														
Emo. exhaustion	0.00	0.02	0.01	0.09*	0.04	0.12	−1.97*	0.03	0.02	0.05	0.00	0.05	0.00	ns
Job competence	0.04	0.03	0.05	−0.04	0.04	−0.06	ns	0.03	0.04	0.02	0.04	0.05	0.05	ns
Stress	−0.00	0.01	−0.02	−0.04***	0.01	−0.21	3.18**	0.00	0.01	0.00	−0.03*	0.02	−0.15	ns
Covariates														
Job satisfaction	0.00	0.03	0.00	−0.03	0.04	−0.04	ns	0.05	0.04	0.05	−0.08	0.05	−0.11	1.98*
Disc. self-efficacy	0.17***	0.04	0.12	0.42***	0.07	0.32	−3.13**	−0.06	0.06	−0.03	0.03	0.08	0.02	ns
Years in ECE	−0.00	0.00	−0.02	0.02	0.02	0.13	ns	0.01	0.01	0.06	0.03	0.02	0.19	ns
BA or higher	−0.25***	0.05	−0.13	−0.37***	0.10	−0.18	ns	−0.02	0.08	−0.01	0.00	0.13	0.00	ns
Health	−0.06	0.04	−0.05	−0.18**	0.06	−0.19	ns	−0.03	0.05	−0.02	−0.13	0.07	−0.12	ns
Age	0.00	0.00	0.02	−0.02	0.01	−0.16	ns	−0.00	0.00	−0.05	0.00	0.01	0.02	ns
Female	0.11	0.13	0.02	−0.53**	0.17	−0.11	3.05**	0.24	0.21	0.03	−0.67**	0.24	−0.12	2.95**
Intercept	4.27***	0.39	4.83	4.45***	0.60	4.90	ns	3.09***	0.50	2.68	4.52***	0.84	4.46	ns

	Negative reactions						
	US			SK			Multi-group
	B	SE	*β*	B	SE	*β*	
Mediators							
Reappraisal	0.00	0.02	0.01	−0.10	0.06	−0.09	ns
Suppression^a^	−0.09***	0.02	−0.17	−0.16**	0.05	−0.19	ns
Psych. wellbeing							
Emo. exhaustion	−0.01	0.01	−0.02	0.11*	0.05	0.13	−2.36*
Job competence	−0.01	0.04	−0.02	−0.14*	0.06	−0.18	ns
Stress	0.01	0.00	0.06	0.05**	0.01	0.20	−2.56*
Covariates							
Job satisfaction	−0.02	0.03	−0.03	0.01	0.06	0.01	ns
Disc. self-efficacy	−0.04	0.03	−0.04	0.16	0.10	0.11	ns
Years in ECE	0.00	0.00	0.03	0.04***	0.01	0.27	−3.32**
BA or higher	0.02	0.04	0.01	−0.19	0.12	−0.08	ns
Health	0.06	0.03	0.07	0.08	0.06	0.07	ns
Age	−0.00	0.00	−0.07	−0.01	0.01	−0.09	ns
Female	−0.38*	0.19	−0.10	0.08	0.19	0.01	ns
Intercept	2.33***	0.34	3.66	2.64***	0.72	2.56	ns

	Reappraisal emotion regulation							Suppression emotion regulation^a^						
	US			SK			Multi-Group	US			SK			Multi-Group
	B	SE	*β*	B	SE	*β*		B	SE	*β*	B	SE	*β*	
Psych. wellbeing														
Emo. exhaustion	0.01	0.02	0.01	0.07	0.05	0.09	ns	−0.05	0.02	−0.07	−0.15**	0.05	−0.15	ns
Job competence	0.07*	0.03	0.07	0.16**	0.06	0.20	ns	0.12**	0.04	0.10	0.08	0.07	0.09	ns
Stress	−0.01	0.01	−0.06	−0.04*	0.01	−0.17	ns	−0.01	0.01	−0.03	−0.06**	0.02	−0.22	2.77**
Covariates														
Job satisfaction	0.08**	0.03	0.09	0.15**	0.06	0.20	ns	0.04	0.03	0.03	−0.25***	0.07	−0.27	3.55***
Disc. self-efficacy	0.22***	0.05	0.14	−0.00	0.10	−0.00	2.06*	−0.05	0.06	−0.03	0.07	0.12	0.04	ns
Years in ECE	−0.01	0.00	−0.08	−0.04***	0.01	−0.23	2.61**	−0.00	0.01	−0.00	−0.00	0.01	−0.01	ns
BA or higher	−0.09	0.07	−0.04	0.46***	0.10	0.21	−4.58***	0.25**	0.08	0.10	0.09	0.16	0.03	ns
Health	0.05	0.04	0.04	0.00	0.06	0.00	ns	0.07	0.05	0.05	0.11	0.10	0.09	ns
Age	0.01**	0.00	0.12	0.03**	0.01	0.25	−2.11*	−0.00	0.00	−0.04	−0.02*	0.01	−0.16	ns
Female	−0.05	0.14	−0.01	−0.81**	0.25	−0.15	2.57*	0.35	0.24	0.05	1.11***	0.25	0.16	ns
Intercept	3.34***	0.39	3.43	3.66***	0.65	3.68	ns	3.65***	0.50	3.12	5.93***	0.80	4.74	−2.19*

B, unstandardized coefficients; SE, bootstrapped standard error; β, standardized coefficients; ns, not significant.

^a^Higher scores refer to lower suppression emotion regulation.

*p < 0.05, **p < 0.01, ***p < 0.001.

**Table 4:** Total, direct, and indirect associations among the study variables.

	Positively-focused reactions							Expressive encouragement						
	US			SK			Multi-group	US			SK			Multi-group
	B	SE	*β*	B	B	*β*		B	SE	*β*	B	SE	*β*	
Emotional exhaustion														
Total	0.01	0.02	0.01	0.11*	0.04	0.15	–	0.03	0.02	0.05	0.01	0.05	0.01	-
Direct	0.00	0.02	0.01	0.09*	0.04	0.12	−3.24**	0.03	0.02	0.05	0.00	0.05	0.00	ns
Indirect	0.00	0.00	0.00	0.02	0.02	0.02	–	−0.00	0.01	−0.00	0.01	0.02	0.01	–
Via reappraisal	0.00	0.00	0.00	0.03	0.02	0.03	ns	0.00	0.01	0.00	0.02	0.01	0.02	ns
Via suppression^a^	0.00	0.00	0.00	−0.01	0.01	−0.01	ns	−0.00	0.00	−0.00	−0.01	0.01	−0.01	ns
Job-related competence														
Total	0.05	0.03	0.06	0.01	0.05	0.02	–	0.05	0.04	0.05	0.09	0.05	0.12	–
Direct	0.04	0.03	0.05	−0.04	0.04	−0.06	ns	0.03	0.04	0.02	0.04	0.05	0.05	ns
Indirect	0.01	0.01	0.01	0.06**	0.02	0.08	–	0.03**	0.01	0.02	0.05**	0.02	0.07	–
Via reappraisal	0.01	0.01	0.01	0.05*	0.02	0.05	ns	0.02	0.01	0.02	0.04*	0.02	0.04	ns
Via suppression^a^	−0.00	0.00	−0.00	0.00	0.00	0.00	ns	0.01*	0.00	0.01	0.01	0.01	0.01	ns
Personal stress														
Total	−0.00	0.01	−0.03	−0.06***	0.01	−0.29	–	−0.00	0.01	−0.01	−0.05**	0.02	−0.22	–
Direct	−0.00	0.01	−0.02	−0.04***	0.01	−0.21	3.18**	0.00	0.01	0.00	−0.03*	0.02	−0.15	4.19*
Indirect	−0.00	0.00	−0.01	−0.01*	0.01	−0.08	–	−0.00*	0.00	−0.02	−0.02**	0.01	−0.07	–
Via reappraisal	−0.00	0.00	−0.00	−0.01*	0.01	−0.01	1.97*	−0.00	0.00	−0.00	−0.01*	0.00	−0.01	ns
Via suppression^a^	0.00	0.00	0.00	−0.00	0.00	−0.00	ns	−0.00	0.00	−0.00	−0.01	0.00	−0.01	ns

	Negative reactions						
	US			SK			Multi-group
	B	SE	*β*	B	SE	*β*	
Emotional exhaustion							
Total	−0.00	0.01	−0.00	0.12**	0.05	0.15	-
Direct	−0.01	0.01	−0.02	0.11*	0.05	0.13	−2.36*
Indirect	0.00	0.00	0.01	0.02	0.01	0.02	–
Via reappraisal	0.00	0.00	0.00	−0.01	0.01	−0.01	ns
Via suppression^a^	0.00	0.00	0.00	0.02*	0.01	0.02	ns
Job-related competence							
Total	−0.02	0.04	−0.04	−0.17**	0.06	−0.21	–
Direct	−0.01	0.04	−0.02	−0.14*	0.06	−0.18	ns
Indirect	−0.01**	0.00	−0.02	−0.03*	0.01	−0.04	–
Via reappraisal	0.00	0.00	0.00	−0.02	0.01	−0.02	ns
Via suppression^a^	−0.01**	0.00	−0.02	−0.01	0.01	−0.01	ns
Personal stress							
Total	0.01	0.01	0.06	0.06***	0.01	0.26	-
Direct	0.01	0.00	0.06	0.05**	0.01	0.20	−2.56*
Indirect	0.00	0.00	0.01	0.01**	0.00	0.06	–
Via reappraisal	−0.00	0.00	−0.00	0.00	0.00	0.02	ns
Via suppression^a^	0.00	0.00	0.00	0.01*	0.00	0.01	−2.10*

B, unstandardized coefficients; SE, bootstrapped standard error; β, standardized coefficients; ns, not significant.

^a^Higher scores refer to lower suppression emotion regulation.

*p < 0.05, **p < 0.01, ***p < 0.001.

First, teachers’ emotional exhaustion, job-related competence, and personal stress were more strongly associated with responsiveness in SK. While none of the direct associations between psychological wellbeing and responsiveness were significant among US teachers, SK teachers displayed substantial associations between wellbeing and responsiveness. The findings indicated significant cross-country differences between the US and SK.

Psychological wellbeing was closely associated with two emotion regulation subscales among teachers in the US and SK. Specifically, US teachers with higher levels of job-related competence tended to use more reappraisal emotion regulation and less suppression emotion regulation. For SK teachers, emotional exhaustion was associated with greater suppression of emotions. Job-related competence was associated with more use of reappraisal emotion regulation. Personal stress was significantly associated with less use of reappraisal emotion regulation and more use of suppression emotion regulation. Among these, only the association between personal stress and suppression emotion regulation was significantly varied by country.

The associations between emotion regulation and responsiveness were also significant across both countries. Reappraisal and suppression were significantly associated with teachers’ emotional responsiveness in the two countries. The association between reappraisal and responsiveness appeared to vary by country. However, there was no significant cross-country variation for associations between suppression emotion regulation and emotional responsiveness.

Next, indirect associations between psychological wellbeing and responsiveness via emotion regulation were examined (Table 4). For US teachers, the indirect associations between job-related competence and responsiveness (i.e., expressive encouragement and negative reactions) appeared to be significant through suppression emotion regulation. When US teachers were competent to their job, they tended to use less suppression emotion regulation, which in turn, was associated with more frequent use of expressive encouragement and fewer use of negative reactions. Meanwhile, SK teachers demonstrated significant indirect associations between all three indicators of psychological wellbeing and responsiveness via either reappraisal or suppression emotion regulation. When SK teachers were emotionally exhausted, they were likely to use more suppression emotion regulation, and ultimately, to use more negative reactions to children. SK teachers’ job-related competence was related to a higher level of reappraisal emotion regulation, which in turn, was associated with more use of expressive encouragement. Furthermore, SK teachers with higher levels of personal stress displayed a lower level of reappraisal emotion regulation, which was then associated with less positively-focused reactions or expressive encouragement. Highly stressed SK teachers also demonstrated more use of suppression emotion regulation, which in turn, was associated with more use of negative reactions. Among these findings, the indirect association between personal stress and positively-focused reactions via reappraisal emotion regulation, and the indirect association between personal stress and negative reactions via suppression emotion regulation were significantly stronger for SK teachers than US teachers.

## Discussion

This study examined indirect associations between psychological wellbeing and emotional responsiveness through emotion regulation among SK and US ECE teachers. In general, significant associations were more prominent among SK teachers, and the associations were also stronger for SK teachers. Moreover, teachers in the two countries displayed different patterns of indirect associations, which potentially indicates that differential use of emotion regulation explains the differences in the associations between teachers’ psychological wellbeing and responsiveness in the US and SK.

As shown in Byun et al. (2022), compared to US teachers, SK teachers reported lower levels of psychological wellbeing, less use of emotion regulation strategies (both reappraisal and suppression), and less desirable patterns of emotional responsiveness. Furthermore, psychological wellbeing and emotion regulation were stronger predictors of teachers’ responsiveness in SK than in the US.

Although US teachers reported no direct associations between all three indicators of wellbeing and emotional responsiveness, US teachers’ job-related competence was directly associated with both increased reappraisal and decreased suppression emotion regulation. This is consistent with what Sheppes (2014) suggested; with more psychological assets (i.e., greater perceived competence in work), teachers are likely to use healthier emotion regulation strategies. Furthermore, US teachers also demonstrated significant associations between emotion regulation and responsiveness. These findings may imply that, in the US, promoting psychological assets and healthy emotion regulation among ECE teachers can potentially be more effective strategies to promote positive teacher practice, rather than focusing on reducing psychological burdens.

As hypothesized, the study found significant indirect associations between psychological wellbeing and responsiveness through emotion regulation in both countries. This finding is meaningful because this is the first study demonstrating the mediating role of emotion regulation between teachers’ wellbeing and practice in ECE settings, which is consistent with findings from other education settings (Moè and Katz, 2021). Similar to the findings on direct associations, indirect associations were more prevalent among SK teachers. Notably, it was often the case that indirect associations were significant via either reappraisal or suppression emotion regulation while no significant direct associations were identified. This trend suggests that, although psychological wellbeing may not be a direct predictor of teachers’ responsiveness, it still plays an important role in their emotional responsiveness toward children via emotion regulation, across both countries. At the same time, emotion regulation deserves greater attention as a target of intervention to improve ECE educators’ positive practice.

Teachers in the US and SK both demonstrated significant indirect associations between job-related competence and psychological wellbeing via emotion regulation. However, the patterns of their indirect associations were substantially different. Specifically, US teachers’ competence in their work was associated with less use of suppression emotion regulation, which was ultimately associated with more desirable emotional responsiveness among teachers. On the contrary, SK teachers’ job-related competence was associated with greater use of reappraisal emotion regulation, which in turn, was associated with more desirable emotional responsiveness. In sum, when teachers in the US and SK were competent in work, they selected differential emotion regulation strategies depending on the national contexts, both of which were associated with higher levels of psychological wellbeing.

This finding illustrates how teachers in different cultural and national settings potentially select different emotion regulation strategies, which might explain the differential underlying mechanism between their wellbeing and responsiveness. In the US, where emotions are considered as individual features and presentness of emotional experiences is encouraged and valued, teachers with higher levels of overall job-related competence may also feel competent about their own emotions experienced in classrooms, and tend not to suppress their emotions. Meanwhile, in SK, where emotions are considered as means to maintain and promote harmonious interpersonal relationships, emotionally competent teachers may actively seek ways to redirect their emotions and modify their behaviors. As a result, either via less use of suppression emotion regulation or more use of reappraisal emotion regulation, emotionally competent teachers in both countries ultimately display more desirable responsiveness toward children’s emotions.

Another interesting finding is that, in the US, only suppression emotion regulation mediated the associations between psychological wellbeing and responsiveness. On the contrary, in SK, both reappraisal and suppression demonstrated significant mediating roles. Matsumoto et al. (2008) indicates that, across various countries, using less suppression emotion regulation is significantly correlated with individuals’ happiness and wellbeing. However, the study also suggests that, in countries valuing perseverance and thrift for future rewards, such as SK, the correlation between reappraisal and suppression emotion regulation appears to be stronger (Matsumoto et al., 2008). It may be the case that the current findings reflect these trends; the role of reappraisal is found to be as prominent as the role of suppression in SK, whereas the role of reappraisal is not evident in the US.

In addition, among our SK teachers, reappraisal and suppression emotion regulation arguably played differential roles. Indirect associations via reappraisal emotion regulation were significant for two positive types of responsiveness, while indirect associations via suppression emotion regulation were significant for negative reactions. In fact, teachers’ reappraisal and suppression emotion regulation have been found to play unique roles in other education settings, too. For instance, in Moè and Katz (2021), high levels of reappraisal was associated with more positive teacher practice, whereas high levels of suppression was associated with less desirable practice. Lockwood et al. (2014) found that suppression emotion regulation was associated with lower levels of cognitive empathy (e.g., perspective taking and cognitive simulation) and affective empathy (e.g., shared emotions), whereas reappraisal emotion regulation was associated with higher levels of cognitive empathy. Suppressing their emotions, SK ECE teachers might have experienced lower levels of empathy for children, thus, they demonstrated more negative reactions to children’s emotions. However, reappraisal emotion regulation may help teachers separate themselves from the influences of emotions so that they can objectively evaluate children’s internal states and provide positive reactions and encouragements.

It is still a concern that the suppression emotion regulation is playing substantial role in indirect associations between wellbeing and responsiveness in both countries. This indicates that, to reduce negative responsiveness, it may be critical to effectively manage suppression emotion regulation. Previous studies suggest that some intervention strategies (e.g., yoga) might be helpful to reduce suppression emotion regulation (e.g., Dick et al., 2014). However, interventions explicitly focusing on educators often found significant effects only on reappraisal emotion regulation, not on suppression emotion regulation (e.g., Hwang et al., 2019). Therefore, while continuing efforts to identify effective strategies to directly reduce suppression emotion regulation among teachers, it might be important to fundamentally remove sources of negative emotions for teachers, for example, challenging work conditions (Jeon and Ardeleanu, 2020).

### Limitations

Despite these important findings, the study has several limitations to be addressed. First, the data used in the study is cross-sectional data, thus, causal interpretation of the findings needs to be avoided. There are possibilities of bidirectional associations between psychological wellbeing and responsiveness among teachers. For example, it may be the case that teachers’ negative responsiveness challenged their psychological-wellbeing, rather than wellbeing influencing teachers’ responsiveness (Friedman-Krauss et al., 2014).

Second, given that all measures of the study are based on teachers’ self-report, the study is susceptible to common method bias and response bias. Thus, it is unsure whether the current variables are true representation of teachers’ psychological and emotional states and their actual practice of responsiveness or not. Moreover, the measures of emotional exhaustion and job-related competence are based on one or two items, not estimating a broader range of items potentially relevant to these features. We used this strategy following a previous study (Buettner et al., 2016), however, the investigation of a latent structure of a large scope of items related to emotional exhaustion and job-related competence may provide a more holistic picture of teachers’ psychological wellbeing. Considering these limitations, the variables included in the study should be interpreted with caution.

Third, because this study is a cross-country comparison study which needs to include identical set of variables in models fitted for each country, some key control variables for each country are omitted. For example, because SK is homogeneous in terms of race and ethnicity (e.g., the proportion of foreign migrants in the population was 2.3% in 2019, and 1.2% on average for the past 20 years; United Nations, 2019), we did not collect data on race ethnicity in SK. Therefore, race and ethnicity of our US sample was also not accounted for in our model. Teachers’ salary was also omitted because there are substantial differences in currency and economics in the two countries, making the absolute salary level incomparable. Teacher qualification was not included as well; given that teachers are required to obtain government-issued teacher certification to work in ECE programs in SK, this question was not asked in SK data collection. We still included teachers’ education level and years of ECE experiences as covariates in the model, which can function as proxies of teachers’ professional background. Because of these limitations due to the nature of the multi-group comparison models, we could only investigate the associations of our variables at the aggregated country level, failing to take into account the within-country variations (e.g., potential variations within the US based on race and ethnicity).

Finally, the data collection periods and sampling methods were different between the US and SK. The US data were collected in 2014 whereas the SK data were collected in 2019. Therefore, the current findings on the cross-country variations may reflect a potential historical within the five-year gap in data collection, not the actual cross-country differences. Furthermore, while US teachers were nationally representative sample recruited by stratified random sampling, SK teachers were recruited using snowball sampling, from regions where generally represent urban metropolitan areas. This indicates that the comparison between the US and SK may not necessarily the comparison between the population characteristics of the two countries. It would rather be the comparison between teachers working in the whole US and urban metropolitan teachers in SK. However, considering that the majority of ECE programs are located in urban metropolitan areas in SK (e.g., Ministry of Health and Welfare, 2019), the findings of the current study may still speak about the common characteristic of ECE teachers in SK.

### Implication and conclusion

This study identified significant direct and indirect associations between psychological wellbeing and responsiveness through emotion regulation, which were more prominent among SK teachers. The cross-country variations in the associations among wellbeing, emotion regulation, and responsiveness suggest that differential policy efforts and intervention strategies are needed for ECE teachers in the US and SK. Specifically, for US teachers, strategies to reduce suppression emotion regulation may be more important than strategies to promote reappraisal emotion regulation. On the other hand, SK teachers may benefit from efforts to simultaneously improve both emotion regulation strategies. While there have been intervention efforts to promote children’s emotion regulation (Hoffmann et al., 2020), teachers’ emotion regulation has received less attention as an area of intervention. Given the importance of emotion regulation in promoting positive teacher practice in ECE classrooms, it may be critical to devote more practical efforts to identify effective strategies to support each different type of emotion regulation among ECE teachers.

In addition, more research is needed to identify the roles of alternative emotion regulation strategies other than reappraisal and suppression emotion regulation. This may be relatively more important for US teachers, as the proportions of variance explained for outcomes were smaller for our US sample than SK sample, which indicates that there may be other factors that would better explain US teachers’ responsiveness. In fact, Chang and Taxer (2021) found that teachers in the US use a wider range of emotion regulation strategies in education settings than reappraisal and suppression. It may be the case that some of the alternative emotion regulation strategies such as situation selection or modification might play significant roles on teacher practice (Gross, 2014). Teachers may self-reflect occasions that they had used negative responsiveness, and try to avoid those situations or modify those situations when they inevitably encounter such events (e.g., Sutton and Harper, 2009). As these alternative emotion regulation strategies are less studied than reappraisal and suppression, future research focusing on the additional features of emotion regulation may reveal innovative findings to support teachers.

Finally, across both countries, it may be important to increase policy efforts to eliminate the sources of negative emotions among teachers to prevent suppression emotion regulation. Kwon et al. (2020) suggest that teachers’ wellbeing can be promoted by creating a positive work climate, providing competitive compensations and benefits, ensuring breaks, reducing physical job demands, and improving physical safety of the workplace. Targeting each of these specific features might be a way to fundamentally enhance ECE teachers’ wellbeing, and subsequently to improve teachers’ positive practice in ECE settings.

## Data availability statement

The datasets presented in this article are not readily available because we have not obtained IRB approval yet for sharing the dataset to public. Aggregated forms of raw findings can be shared. Requests to access the datasets should be directed to SB, sbyun@virginia.edu.

## Ethics statement

The studies involving human participants were reviewed and approved by the Johns Hopkins University Homewood Institutional Review Board. The patients/participants provided informed consent to participate in this study by completing and returning an anonymous survey.

## Author contributions

SB and LJ contributed to conception and design of the study. SB performed the statistical analysis. SB wrote the first draft of the manuscript. All authors contributed to manuscript revision, read, and approved the submitted version.

## Funding

This study was supported by the Johns Hopkins University School of Education Dean’s Office.

## Conflict of interest

The authors declare that the research was conducted in the absence of any commercial or financial relationships that could be construed as a potential conflict of interest.

## Publisher’s note

All claims expressed in this article are solely those of the authors and do not necessarily represent those of their affiliated organizations, or those of the publisher, the editors and the reviewers. Any product that may be evaluated in this article, or claim that may be made by its manufacturer, is not guaranteed or endorsed by the publisher.
